# Associations between *ALDH* Genetic Variants, Alcohol Consumption, and the Risk of Nasopharyngeal Carcinoma in an East Asian Population

**DOI:** 10.3390/genes12101547

**Published:** 2021-09-29

**Authors:** Wen-Ling Liao, Fu-Chun Chan, Kai-Ping Chang, Ya-Wen Chang, Che-Hong Chen, Wen-Hui Su, Hen-Hong Chang

**Affiliations:** 1 Graduate Institute of Integrated Medicine, College of Chinese Medicine, China Medical University, Taichung 40402, Taiwan; wl0129@mail.cmu.edu.tw (W.-L.L.); windy0803@gmail.com (Y.-W.C.); 2 Center for Personalized Medicine, China Medical University Hospital, Taichung 40447, Taiwan; 3 Graduate Institute of Biomedical Sciences, College of Medicine, Chang Gung University, Taoyuan 33302, Taiwan; fugi8396@gmail.com; 4 Department of Otolaryngology—Head & Neck Surgery, Chang Gung Memorial Hospital, Linkou Medical Center, Taoyuan 33302, Taiwan; dr.kpchang@gmail.com; 5 Human Genetic Center, Department of Medical Research, China Medical University Hospital, China Medical University, Taichung 40402, Taiwan; 6 Department of Chemical and Systems Biology, Stanford University School of Medicine, Stanford, CA 94305, USA; chehong@stanford.edu; 7 Department of Biomedical Sciences, College of Medicine, Chang Gung University, Taoyuan 33302, Taiwan; 8 Chang Gung Molecular Medicine Research Center, Chang Gung University, Taoyuan 33302, Taiwan; 9 Department of Chinese Medicine, China Medical University Hospital, Taichung 40402, Taiwan; 10 Traditional Chinese Medicine Research Center, China Medical University, Taichung 40402, Taiwan

**Keywords:** nasopharyngeal carcinoma, aldehyde dehydrogenase, single nucleotide polymorphism, alcohol

## Abstract

Nasopharyngeal carcinoma (NPC) and alcohol flush syndrome are thought to be strongly influenced by genetic factors and are highly prevalent amongst East Asians. Diminished activity of aldehyde dehydrogenase (ALDH), a major enzyme in the alcohol-metabolizing pathway, causes the flushing syndrome associated with alcoholic consumption. The genetic effect of *ALDH* isoforms on NPC is unknown. We therefore investigated the association between the genetic polymorphisms of all 19 *ALDH* isoforms and NPC among 458 patients with NPC and 1672 age- and gender-matched healthy controls in Taiwan. Single-nucleotide polymorphisms (SNPs) located between the 40,000 base pairs upstream and downstream of the 19 *ALDH* isoform coding regions were collected from two genome-wise association studies conducted in Taiwan and from the Taiwan Biobank. Thirteen SNPs located on *ALDH4A1*, *ALDH18A1*, *ALDH3B2*, *ALDH1L2, ALDH1A2,* and *ALDH2* Glu487Lys (rs671) were associated with NPC susceptibility. Stratification by alcohol status revealed a cumulative risk effect for NPC amongst drinkers and non-drinkers, with odds ratios of 4.89 (95% confidence interval 2.15–11.08) and 3.57 (1.97–6.47), respectively. A synergistic effect was observed between SNPs and alcohol. This study is the first to report associations between genetic variants in 19 *ALDH* isoforms, their interaction with alcohol consumption and NPC in an East Asian population.

## 1. Introduction

Nasopharyngeal carcinoma (NPC) is more common in East and Southeast Asia than in Western countries, with more than 70% of cases worldwide originating from this Asian region. Recently reported age-standardized incidence rates range from 3.0 per 100,000 in China to 0.4 per 100,000 in Western countries [1,2]. The remarkable geographical distribution of NPC incidence and family history as a strong risk factor suggests that host genetic susceptibility plays an important role [2,3]. Familial linkage studies, genetic case-control association studies, genome-wide association studies (GWAS) and whole-exome sequencing association studies have identified susceptibility genes/loci related to the risk of NPC, including the *HLA* genes [4,5], *CLPTM1L/TERT* [6], *MST1R* [7] and *NIPAL1* genes [3]. Additional host genetic susceptibility factors have remained elusive.

Lifestyle behaviors such as salted fish intake and cigarette smoking significantly increase the risk of NPC in Asian populations [8]. The association between alcohol consumption and NPC risk is inconsistent in many studies [9,10]. Two meta-analyses have indicated that the risk of developing NPC may increase with alcohol consumption; in both meta-analyses, drinking and high-frequency drinking increased the risk of NPC [11,12].

People of East Asian descent have the highest prevalence (35–45%) of aldehyde dehydrogenase 2 (ALDH2) deficiency, which causes the flushing syndrome observed after consumption of alcoholic beverages [13]. The major alcohol-metabolizing enzymes are alcohol dehydrogenase-1B (ADH1B) and ALDH2, and the two most frequently reported polymorphisms, *ADH1B* Arg47His (rs1229984) and *ALDH2* Glu487Lys (rs671), have been shown to alter the effect of alcohol and potentially influence carcinogenesis [14,15]. In humans, the ALDH family consists of 19 members identified through similar amino acid sequences and functions [16]. Several recent studies have shown that *ALDH1A1*, *ALDH2* and *ALDH3A1* may be related to different cancers, such as head and neck cancer (HNC) [17], esophageal cancer [18], cholangiocarcinoma [19], and colorectal cancer (CRC) [14]. Elevated *ALDH1* activity has been used as a cancer stem cell biomarker of tumor aggressiveness in the invasive front of NPC [20], while a lower expression of *ALDH2* has been associated with poor prognoses in breast cancer, lung adenocarcinoma, and HNC squamous cell carcinomas [21]. 

Although NPC and *ALDH2* deficiency occur with high frequencies among East Asians, the genetic effects of *ALDH* isoforms on NPC remain unknown. Thus, we sought to determine associations between the genetic polymorphisms of 19 *ALDH* isoforms, [22] their interaction with alcohol consumption and NPC in an Asian population in Taiwan.

## 2. Materials and Methods

### 2.1. Study Population 

This study included 458 NPC cases enrolled in two GWAS studies conducted in Taiwan [4,23], all of whom were recruited from Chang Gung Memorial Hospital (CGMH) between 1983 and 2008. Their pathology records were reviewed for confirmation of NPC diagnosis according to World Health Organization (WHO) pathological classification criteria. Age- and gender-matched healthy controls were randomly selected from subjects without any NPC family history from the Taiwan Biobank (TWB) [24]. After matching, a total of 413 NPC cases and 1672 healthy controls were included in the present study (case:control ratio 1:4). The TWB has collected specimens and associated data (including genetic information) from the general Taiwanese population since 2013 and follows up with subjects every two to four years. The TWB data in this study involved individuals aged 30–70 years who self-reported as being of Taiwanese Han Chinese descent. The study was reviewed and approved by the Institutional Review Broad of Chang Gung Medical Foundation, Taiwan (IRB 103-7224B). Written informed consent was obtained from each study participant at the time of enrollment.

### 2.2. Data Collection

Survey questionnaires collected information about alcohol consumption, betel quid chewing, and cigarette smoking. Cases were designated alcohol users if they had consumed an alcoholic beverage at least once weekly for six months, betel nut users if they had chewed at least two betel nuts daily for a year, and cigarette smokers if they had smoked daily for at least one year. Among controls, alcohol users were defined as persons who reported drinking more than 150 mL of alcohol per week during the 6 months before the study health examination, betel nut users if they had ever chewed betel nuts daily for one month, and cigarette smokers if they had smoked daily for at least 6 months. 

### 2.3. Genotyping and Imputation 

Genotyping of the NPC cohort was performed by Illumina Hap550v3_A (for 277 NPC cases) and Human610-Quad Beadchips (for 181 NPC cases), according to the manufacturer’s protocols (Illumina, Inc., San Diego, CA, USA). The Affymetrix Axiom genome-wide TWB array was used to genotype the TWB cohort. Genotyping and quality control measures involving samples and single nucleotide polymorphisms (SNPs) followed those described in previous studies [4,23]. Since the GWAS results were obtained using three different genotyping platforms, genotype imputations were performed separately in each platform before data combination. Imputations were performed using IMPUTE2 [25] with the 1000 Genomes Project Phase III reference panel (October 2014 release). A total of 78,605 SNPs were identified between the 20,000 base pairs upstream and 20,000 base pairs downstream of 19 *ALDH* isoform coding regions, based on GENCODE release 38. SNPs with low imputation quality (information < 0.3), call rate < 99%, minor allele frequency < 0.05, and Hardy-Weinberg equilibrium in controls (*p* < 0.0005) were removed from analysis.

### 2.4. Statistical Analysis

For the baseline characteristics, continuous data are presented as means with standard deviation, and categorical data are presented as proportions. We used *t*-tests to compare mean values of continuous variables and chi-squared tests to compare the frequencies of categorical variables between two groups. The association between SNP genotype/cumulative risk alleles and disease status was evaluated using logistic regression while controlling for alcohol use, betel quid chewing, and cigarette smoking to obtain the *p* values, odds ratios (ORs) and 95% confidence intervals (CIs) in PLINK (version 1.90) [26]. Permutation testing was performed 10,000 times using the PLINK “-mperm 10000” command. All tests were two-sided, and a *p* value < 0.05 was considered to be statistically significant. Statistical analyses were performed using SPSS software v21.0 for Windows (IBM, Armonk, NY, USA) and R version 3.4.4 (R Core Team, 2018).

## 3. Results

### 3.1. Characteristics of the Study Participants

**Figure 1:**
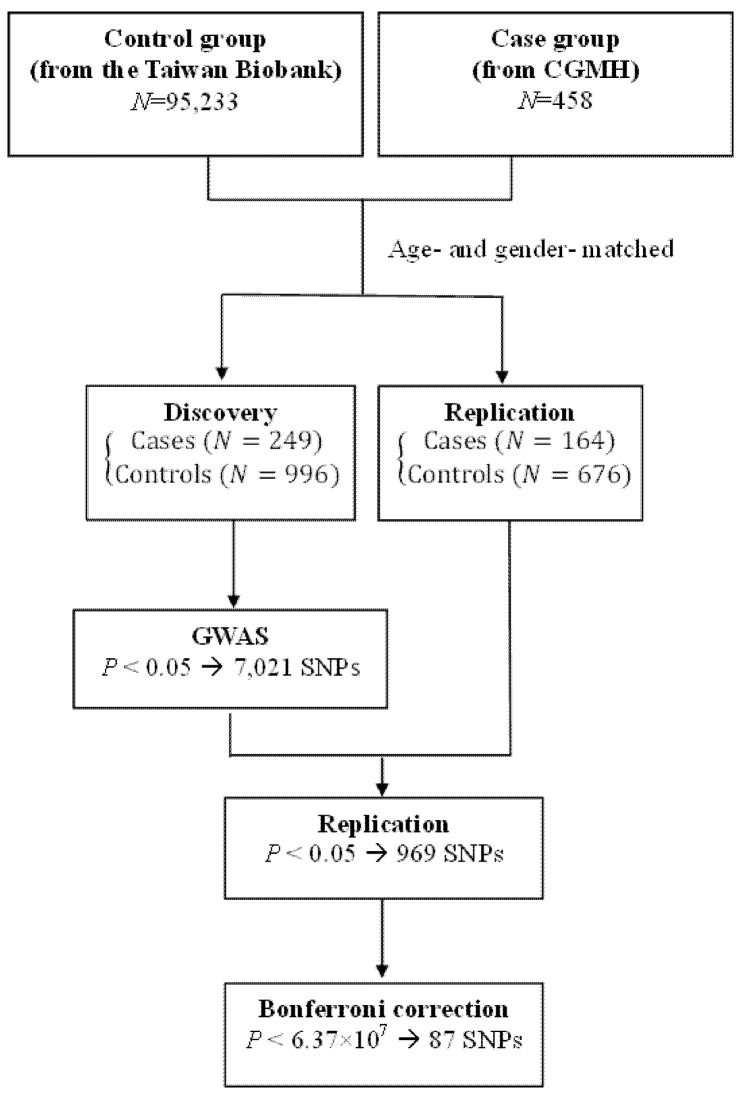
Flow chart of the study design.

A total of 1245 subjects (249 cases and 996 controls) served as the discovery cohort to search for genetic risk factors associated with NPC, while 840 subjects (164 cases and 676 controls) served as the replication cohort for the identified genetic SNPs (Figure 1). Demographic characteristics of patients and controls are presented in Table 1. Around three-quarters (75%) of the study population were males; mean ages were 47.98 ± 10.03 years in the NPC group and 48.03 ± 10.37 years in the control group. Significantly higher proportions of the NPC group consumed alcohol, chewed betel quid, and smoked cigarettes, compared with the controls (36.8% vs. 15.6%; 23.0% vs. 6.0%; and 48.9% vs. 35.1%, respectively; all *p* values < 0.001). Around two-thirds of the NPC cases (67.5%) were diagnosed with late-stage (III and IV) disease, as according to the WHO classification (data not shown). Clinical characteristics including alcohol use, betel quid chewing, and cigarette smoking are risk factors for NPC and were included in the subsequent adjusted genetic SNP analysis.

### 3.2. ALDH Isoforms and Candidate SNPs Confer Susceptibility for NPC

We determined the association between the genetic polymorphisms of 19 *ALDH* isoforms and the risk of NPC in Taiwan Chinese. Multivariate logistic regression analysis adjusted for alcohol drinking, betel quid chewing, and cigarette smoking identified 12 SNPs on *ALDH4A1*, *ALDH18A1*, *ALDH3B2*, *ALDH1L2*, and *ALDH1A2* that were significantly associated with an increased risk for NPC (all *p* values < 0.05, Table 2). In this study, we used the permutation test, a robust but computationally intensive alternative to the conservative Bonferroni correction for correcting multiple testing [27]. Although none of the SNPs remained significant after Bonferroni correction (0.05/78,605), 6 SNPs located in *ALDH4A1*, *ALDH18A1* and *ALDH3B2* passed a 10,000 random shuffled permutation test (*p* perm < 0.05). In particular, one SNP (rs7534676) located in *ALDH4A1* had a significant permutation *p* value of <0.01 (Table 2). 

This study also investigated the two most frequently reported gene polymorphisms related to alcohol metabolism, *ADH1B* Arg47His rs1229984 and *ALDH2* Glu487Lys rs671. An association was observed between the rs671 polymorphism in *ALDH2* and NPC risk. The adjusted OR was 1.23 (95% CI = 1.03–1.48, *p* = 0.00225) when increased by one A allele. No association was observed between the rs1229984 polymorphism in *ADH1B* and NPC risk. After adjusting for potential confounders, the OR was 0.97 (95% CI = 0.78–1.22, *p* = 0.0801) (Table 2).

### 3.3. Cumulative Risk Effect of 13 SNPs on NPC Susceptibility

Total risk allele counts for the 13 SNPs that we have identified were calculated for each subject (range 13–26; median 23). In the multivariate logistic regression model, alcohol use, betel nut chewing and the cumulative risk allele were all independent risk factors for NPC. ORs were 2.61 (95% CI = 1.60–4.26, *p* < 0.001) for alcohol use and 2.63 (1.40–4.94, *p* = 0.003) for betel nut chewing. Study subjects with more than 23 risk alleles had a significantly higher risk of NPC (OR = 3.98; 95% CI = 2.45–6.46, *p* < 0.001) compared with subjects with fewer than 23 risk alleles (Table 3).

### 3.4. ALDH Genes Confer Susceptibility for NPC after Stratification for Alcohol Use

To investigate the confounding effect of alcohol use on NPC, associations between SNPs and NPC were stratified by alcohol consumption. Among subjects who did not consume alcohol, the homozygous risk allele for most SNPs (except rs1229984) increased the risk of NPC (*p* < 0.05). Among alcohol users, the homozygous risk alleles for rs7534676, rs7554974, rs7518631, rs7518631, rs72936453, rs1711068, rs76655136, rs1975431, and rs28829404 increased the risk of NPC (*p* < 0.05). For *ALDH2* rs671, the risk of NPC was higher for study subjects with the AA/AG alleles compared with subjects with the GG allele, whether alcohol was consumed (OR = 1.47; 95% CI = 0.95–2.27, *p* = 0.082) or not (1.27; 0.97–1.67, *p* = 0.087). A cumulative risk allele effect for NPC was observed with alcohol consumption: the risk was lower for subjects not using alcohol (OR = 3.57; 95% CI = 1.97–6.47, *p* < 0.001) than for those who were (4.89; 2.15–11.08, *p* < 0.001) (Table 4).

### 3.5. The Effects of Interaction between Alcohol Consumption and SNPs on the Risk of NPC

An investigation into the effects of interaction between alcohol consumption and SNPs on the risk of NPC revealed that the risk increases with either the presence of a risk allele or alcohol consumption. For the rs671 polymorphism, the NPC risk was significantly increased among AA/AG carriers who were not consuming alcohol or GG carriers who were consuming alcohol (OR = 1.63; 95% CI = 1.27–2.10, *p* < 0.001), and AA/AG carriers who were consuming alcohol (4.55; 3.02–6.84, *p* < 0.001), compared with carriers of the GG genotype who did not consume alcohol (Table 5).

## 4. Discussion

To the best of our knowledge, this study is the first to investigate the association between genetic variants in 19 *ALDH* isoform polymorphisms and the risk of NPC in an East Asian population residing in Taiwan. Besides the known alcohol metabolism genetic variant, rs671, we identified 12 SNPs located on the *ALDH4A1*, *ALDH18A1*, *ALDH3B2*, *ALDH1L2*, and *ALDH1A2* genes from the *ALDH* multigene family that were associated with an elevated NPC risk. 

ALDHs are a family of intracellular enzymes that are involved in aldehyde metabolism, cellular detoxification, differentiation, and cancer drug [28,29]. Several isoforms of the *ALDH1* family (*ALDH1A1*, *ALDH1A2*, *ALDH1A3*, *ALDH1B1*, *ALDH1L1,* and *ALDH1L2*) are used as cancer stem cell markers in a variety of cancers [29,30,31]. Strong correlations between *ALDH1* expression in the invasive tumor front of NPC, epithelial-mesenchymal transition (EMT) and tumor aggressiveness suggest that *ALDH1* expression in the invasive front of NPC could be a useful prognostic marker for NPC patients [20]. RNA sequencing data from The Cancer Genome Atlas (TCGA) database have revealed downregulated *ALDH1A2* and *ALDH1L1* expression in esophageal squamous cell carcinoma and HNC squamous cell carcinoma [21]. Meta-analysis results found that lower *ALDH1A1* and *ALDH1L1* expression was associated with poorer overall survival and poorer progression-free survival in cancer patients [21]. In our study, SNPs located on the *ALDH1L2* and *ALDH1A2* genes were associated with the risk of developing NPC. Decreased levels of *ALDH1A1*, *ALDH1A2*, *ALDH1A3,* and *ALDH1L1* expression were observed in 5 pairwise samples of nasopharynx squamous cell carcinoma (the results are not shown). 

Polymorphisms in genes responsible for the alcohol metabolism pathways can affect the amount of acetaldehyde and reactive oxygen species generated during the metabolic process, and thus alter the effects of alcohol and potentially influence carcinogenesis [14,15]. *ADH1B* Arg47His (rs1229984) and *ALDH2* Glu487Lys (rs671) are the most frequently reported genetic polymorphisms related to alcohol metabolism. Both variants are not only related to alcohol metabolism but also to cancer risk. A 40-fold decrease in *ADH1B* activity has been observed in *ADH1B* His/His individuals [32], while a loss of ALDH2 enzyme activity has been observed in individuals with the *ALDH2* Lys/Lys phenotype [14,33]. Many studies have demonstrated that the genetic effect of *ADH1B* and *ALDH2* increase the risk of different types of cancers [34]. However, SNP rs1229884 in the *ADH1B* gene was not significantly associated with NPC in our Han Chinese patients in Taiwan, which is consistent with the results from previous meta-analyses showing that the *ALDH2* polymorphism, but not the *ADH1B* polymorphism, significantly increases the risk of CRC in East Asians [14,35]. 

Other research has reported that heavy alcohol consumption can increase the risk of certain cancer types, including HNC cancers and NPC [11]. In studies involving East Asian populations, the presence of genetic polymorphisms in *ADH1B* (rs1229984) and *ALDH2* (rs671), as well as alcohol consumption, individually or in combination [13], increase the risk of breast cancer [36], HNC [17], and esophageal cancer [18,35]. Moreover, research has shown that alcohol consumption affects two major folate-metabolizing enzymes, ALDH1L1 and ALDH1L2, with a possible synergistic effect on carcinogenesis [37,38]. In this study, SNPs rs671 located on *ALDH2* and rs10778364 located on *ALDH1L2* were significantly associated with an increased risk for NPC, with or without alcohol consumption. We also observed a synergistic effect between SNPs and alcohol consumption. These findings indicate that not only alcohol plays a role in the risk of NPC, but that the genetic effects of *ALDH2* and *ALDH1L2* are also important for NPC risk. 

Inconsistent associations for alcohol consumption, betel nut chewing, and tobacco smoking have been recorded in previous studies [8,12]. This inconsistency may be due to differences in study populations, NPC subtypes, or definitions of lifestyle behaviors. A significant association between alcohol intake and NPC risk was observed in this study and other research [39,40,41], while several studies have observed a lack of association between alcohol and NPC risk [10,42,43]. We also observed that betel nut chewing was significantly associated with NPC risk. Although three previous studies found no such association [44], a positive association has been reported between betel nut chewing and NPC risk in NPC high-risk families in Taiwan [45]. A modestly increased risk of NPC associated with tobacco smoking has been reported in southern China [46], which is consistent with our study.

This study is apparently the first to discuss associations between the genetic variants of 19 *ALDH* isoforms and NPC. However, some limitations in this study must be noted. First, due to the low frequency of alcohol consumption and low frequency of risk alleles, the numbers in each subgroup for SNPs and alcohol interactions are small and the statistical power is limited. Second, recall bias may exist, since the information about alcohol, betel quid chewing, and cigarette smoking was collected by self-reported questionnaires. Third, selection bias may exist, since the NPC cases and controls were enrolled under different projects (a hospital for the NPC cases, whereas controls were recruited from communities throughout Taiwan). Fourth, different measurement scales used for alcohol consumption, betel nut chewing, and cigarette smoking in these two projects may have led to misclassification.

## 5. Conclusions

In conclusion, our data demonstrate that the risk of NPC is increased in the presence of genetic variants of different ALDH isoforms. The potential of using genetic variants of *ALDH* as biomarkers to help to identify potential screening populations for NPC awaits future investigations.

## Figures and Tables

**Table 1:** Demographics of the study population.

	Database 1-Discovery (*N* = 1245)			Database 2-Replication (*N* = 840)			Database 3-Total (*N* = 2085)		
	Controls (*N* = 996)	Cases (*N* = 249)	*p* Value	Controls (*N* = 676)	Cases (*N* = 164)	*p* Value	Controls (*N* = 1672)	Cases (*N* = 413)	*p* Value
Age	48.16 (9.92)	48.01 (9.43)	0.827	47.84 (11.00)	47.92 (10.91)	0.934	48.03 (10.37)	47.98 (10.03)	0.919
Gender			1.000			0.924			0.939
Female	228 (22.9)	57 (22.9)		188 (27.8)	45 (27.4)		416 (24.9)	102 (24.7)	
Male	768 (77.1)	192 (77.1)		488 (72.2)	119 (72.6)		1256 (75.1)	311 (75.3)	
Alcohol users			<0.001 **			<0.001 **			<0.001 **
No	830 (83.4)	156 (62.7)		581 (85.9)	105 (64.0)		1411 (84.4)	261 (63.2)	
Yes	165 (16.6)	93 (37.3)		95 (14.1)	59 (36.0)		260 (15.6)	152 (36.8)	
Betel quid chewers			<0.001 **			<0.001 **			<0.001 **
No	937 (94.3)	189 (75.9)		631 (93.6)	129 (78.7)		1568 (94.0)	318 (77.0)	
Yes	57 (5.7)	60 (24.1)		43 (6.4)	35 (21.3)		100 (6.0)	95 (23.0)	
Cigarette smokers			0.005 *			<0.001 **			<0.001 **
No	620 (62.3)	131 (52.6)		464 (68.6)	80 (48.8)		1084 (64.9)	211 (51.1)	
Yes	375 (37.7)	118 (47.4)		212 (31.4)	84 (51.2)		587 (35.1)	202 (48.9)	

Values are presented as *N* (%) or mean (SD). *p* values for chi square test or two independent *t*-tests: * represent *p* values less than 0.05; ** represent *p* values less than 0.001.

**Table 2:** Associations between *ALDH* gene polymorphisms and nasopharyngeal carcinoma risk.

rsID	Genes	Chr.	pb38	Risk Allele	Discovery	Replication	Total			
					OR (95% CI)	OR (95% CI)	Genotype Frequency		OR (95% CI)	*p* Value
							Cases	Controls		
rs7534676	*ALDH4A1;RP13-279N23.2*	1	18,893,311	C	0.35 (0.2–0.62)	0.34 (0.18–0.65)	3/20/320	16/294/1336	0.35 (0.23–0.53)	8.92 × 10^7^ *
rs7554974	*ALDH4A1;RP13-279N23.2*	1	18,897,992	T	0.41 (0.25–0.69)	0.34 (0.18–0.64)	3/23/323	17/299/1340	0.38 (0.26–0.57)	2.13 × 10^6^ *
rs7518631	*ALDH4A1;RP13-279N23.2*	1	18,899,249	A	0.43 (0.26–0.72)	0.34 (0.18–0.64)	3/24/322	18/299/1339	0.40 (0.27–0.58)	3.24 × 10^6^ *
rs72936453	*ALDH4A1;RP13-279N23.2*	1	18,899,852	T	0.43 (0.26–0.72)	0.34 (0.18–0.63)	3/24/323	18/299/1339	0.39 (0.27–0.58)	3.01 × 10^6^ *
rs1229984	*ADH1B*	4	99,318,162	T	0.95 (0.70–1.30)	1.06 (0.76–1.50)	16/86/132	104/656/912	1.00 (0.79–1.25)	9.72 × 10^1^
rs17111068	*ALDH18A1*	10	95,638,799	T	0.12 (0.04–0.38)	0.12 (0.03–0.49)	0/5/304	6/204/1462	0.12 (0.05–0.30)	3.91 × 10^6^ *
rs76655136	*ALDH3B2;RP11-119D9.1*	11	67,695,970	C	0.14 (0.05–0.38)	0.37 (0.17–0.83)	0/11/333	11/191/1470	0.23 (0.12–0.42)	3.02 × 10^6^ *
rs10778364	*ALDH1L2;C12orf45*	12	105,047,085	T	0.68 (0.52–0.89)	0.66 (0.47–0.94)	8/115/246	96/598/960	0.67 (0.54–0.83)	1.93 × 10^4^
rs671	*ALDH2*	12	11,180,3962	A	1.32 (1.05–1.67)	1.10 (0.82–1.47)	39/163/191	146/684/842	1.23 (1.03–1.48)	2.25 × 10^2^
rs34200934	*RP11-344A16.2;ALDH1A2*	15	57,767,226	T	0.70 (0.50–0.97)	0.66 (0.44–1.01)	8/66/307	30/414/1185	0.69 (0.53–0.89)	4.76 × 10^3^
rs11636446	*RP11-344A16.2;ALDH1A2*	15	57,780,522	C	0.67 (0.46–0.98)	0.65 (0.42–1.01)	3/60/315	21/364/1282	0.67 (0.50–0.89)	5.41 × 10^3^
rs79071218	*RP11-344A16.2;ALDH1A2*	15	57,885,374	C	0.19 (0.08–0.48)	0.41 (0.18–0.93)	0/12/356	4/189/1479	0.28 (0.15–0.51)	3.50 × 10^5^
rs1975431	*RP11-344A16.2;ALDH1A2*	15	57,887,256	C	0.19 (0.08–0.48)	0.36 (0.15–0.87)	0/11/356	4/188/1480	0.26 (0.14–0.48)	2.33 × 10^5^
rs28829404	*ALDH1A2;LIPC*	15	58,379,641	T	0.40 (0.22–0.73)	0.26 (0.09–0.71)	1/14/346	6/196/1470	0.34 (0.20–0.58)	5.32 × 10^5^

Abbreviations: OR: odds ratio; CI: confidence interval. * *p* value for permutation < 0.05.

**Table 3:** Cumulative risk effect of 13 SNPs on nasopharyngeal carcinoma susceptibility in multivariate logistic regression model.

	OR (95% CI)	*p* Value
Alcohol consumption	2.61 (1.60–4.26)	<0.001 **
Betel quid chewing	2.63 (1.40–4.94)	0.003 *
Cigarette smoking	0.79 (0.49–1.27)	0.334
Cumulative risk allele		
<median	Ref.	Ref.
≥median	3.98 (2.45–6.46)	<0.001 **

Abbreviations: OR: odd ratio; CI: confidence interval. The median number of cumulative risk alleles was 23. * *p* value < 0.05; ** *p* value < 0.001.

**Table 4:** Associations between *ALDH* genes and nasopharyngeal carcinoma susceptibility after stratification for alcohol use.

	Alcohol Use = No				Alcohol Use = Yes			
	Controls (*N* = 1411)	Cases (*N* = 261)	OR (95% CI)	*p* Value	Controls (*N* = 260)	Cases (*N* = 152)	OR (95% CI)	*p* Value
rs1229984								
CC/CT	647 (45.9)	68 (43.3)	Ref.	Ref.	112 (43.1)	34 (44.2)	Ref.	Ref.
TT	764 (54.1)	89 (56.7)	1.11 (0.80–1.55)	0.544	148 (56.9)	43 (55.8)	0.96 (0.57–1.60)	0.867
rs671								
GG/AG	1270 (90.0)	211 (84.5)	Ref.	Ref.	255 (98.1)	142 (100.0)	Ref.	Ref.
AA	141 (10.0)	39 (15.5)	1.66 (1.13–2.43)	0.010 *	5 (1.9)	0 (0.0)	0.00 (0.00–)	0.999
Recessive (AA/AG)	755 (53.5)	149 (59.4)	1.27 (0.97–1.67)	0.087	75 (28.8)	53 (37.3)	1.47 (0.95–2.27)	0.082
rs7534676								
TT/TC	253 (18.2)	16 (7.3)	Ref.	Ref.	56 (21.8)	7 (5.6)	Ref.	Ref.
CC	1135 (81.8)	203 (92.7)	2.83 (1.67–4.79)	<0.001 *	201 (78.2)	117 (94.4)	4.66 (2.06–10.55)	<0.001 *
rs7554974								
CC/CT	259 (18.5)	19 (8.5)	Ref.	Ref.	56 (21.8)	7 (5.6)	Ref.	Ref.
TT	1139 (81.5)	205 (91.5)	2.45 (1.51–4.00)	<0.001 *	201 (78.2)	118 (94.4)	4.70 (2.07–10.64)	<0.001 *
rs7518631								
GG/GA	260 (18.6)	19 (8.5)	Ref.	Ref.	56 (21.8)	8 (6.3)	Ref.	Ref.
AA	1138 (81.4)	204 (91.5)	2.45 (1.51–4.00)	<0.001 *	201 (78.2)	118 (93.7)	4.11 (1.89–8.92)	<0.001 *
rs72936453								
AA/AT	260 (18.6)	19 (8.5)	Ref.	Ref.	56 (21.8)	8 (6.3)	Ref.	Ref.
TT	1138 (81.4)	204 (91.5)	2.45 (1.51–4.00)	<0.001 *	201 (78.2)	119 (93.7)	4.14 (1.91–8.99)	<0.001 *
rs17111068								
GG/GT	178 (12.6)	4 (2.0)	Ref.	Ref.	32 (12.3)	1 (0.9)	Ref.	Ref.
TT	1233 (87.4)	195 (98.0)	7.04 (2.58–19.18)	<0.001 *	228 (87.7)	109 (99.1)	15.30 (2.06–113.42)	0.008 *
rs76655136								
TT/TC	167 (11.8)	6 (2.7)	Ref.	Ref.	35 (13.5)	5 (4.1)	Ref.	Ref.
CC	1244 (88.2)	215 (97.3)	4.81 (2.10–11.00)	<0.001 *	225 (86.5)	118 (95.9)	3.67 (1.40–9.62)	0.008 *
rs10778364								
CC/CT	586 (42.0)	76 (32.8)	Ref.	Ref.	107 (41.3)	47 (34.3)	Ref.	Ref.
TT	808 (58.0)	156 (67.2)	1.49 (1.10–2.00)	0.008 *	152 (58.7)	90 (65.7)	1.35 (0.88–2.07)	0.174
rs34200934								
GG/GT	365 (26.6)	42 (17.1)	Ref.	Ref.	79 (30.9)	32 (23.7)	Ref.	Ref.
TT	1007 (73.4)	204 (82.9)	1.76 (1.24–2.51)	0.002 *	177 (69.1)	103 (76.3)	1.44 (0.89–2.32)	0.137
rs11636446								
AA/AC	321 (22.8)	36 (15.2)	Ref.	Ref.	64 (24.7)	27 (19.1)	Ref.	Ref.
CC	1086 (77.2)	201 (84.8)	1.65 (1.13–2.40)	0.009 *	195 (75.3)	114 (80.9)	1.39 (0.84–2.30)	0.206
rs79071218								
TT/TC	170 (12.0)	7 (3.1)	Ref.	Ref.	23 (8.8)	5 (3.5)	Ref.	Ref.
CC	1241 (88.0)	219 (96.9)	4.29 (1.99–9.25)	<0.001 *	237 (91.2)	137 (96.5)	2.66 (0.99–7.15)	0.053
rs1975431								
AA/AC	169 (12.0)	7 (3.1)	Ref.	Ref.	23 (8.8)	4 (2.8)	Ref.	Ref.
CC	1242 (88.0)	219 (96.9)	4.26 (1.97–9.19)	<0.001 *	237 (91.2)	137 (97.2)	3.32 (113–9.81)	0.030 *
rs28829404								
CC/CT	163 (11.6)	13 (5.7)	Ref.	Ref.	39 (15.0)	2 (1.5)	Ref.	Ref.
TT	1248 (88.4)	216 (94.3)	2.17 (1.21–3.89)	0.009 *	221 (85.0)	130 (98.5)	11.47 (2.73–48.29)	0.001 *
Cumulative risk alleles								
<Median	615 (46.3)	14 (19.4)	Ref.	Ref.	142 (56.6)	8 (21.1)	Ref.	Ref.
≥Median	713 (53.7)	58 (80.6)	3.57 (1.97–6.47)	<0.001 *	109 (43.4)	30 (78.9)	4.89 (2.15–11.08)	<0.001 *

Abbreviations: OR: odds ratio; CI: confidence interval. Odds ratios (ORs) were calculated by unadjusted univariate analysis. The median number of cumulative risk alleles was 23. * *p* value < 0.05.

**Table 5:** Effects of interactions between SNPs and alcohol consumption on nasopharyngeal carcinoma risk.

	Controls (*N* = 1672)	Cases (*N* = 413)	*p* Value ^a^	OR (95% CI)	*p* Value ^b^
rs1229984*Alcohol			<0.001		
CC/CT*Non-Alcohol	647 (38.7)	68 (29.1)		Ref.	Ref.
TT*Non-Alcohol or CC/CT*Alcohol	876 (52.4)	123 (52.6)		1.34 (0.98–1.83)	0.070
TT*Alcohol	148 (8.9)	43 (18.4)		2.76 (1.81–4.21)	<0.001 *
rs671*Alcohol			-		
GG/AG*Non-Alcohol	1270 (76.0)	212 (53.9)		Ref.	Ref.
AA*Non-Alcohol or GG/AG*Alcohol	396 (23.7)	181 (46.1)		2.74 (2.18–3.44)	<0.001 *
AA*Alcohol	5 (0.3)	0 (0.0)		- (–)	0.999
(Recessive model)			<0.001		
GG*Non-Alcohol	656 (39.3)	102 (26.0)		Ref.	Ref.
AA/AG*Non-Alcohol or GG*Alcohol	940 (56.3)	238 (60.6)		1.63 (1.27–2.10)	<0.001 *
AA/AG*Alcohol	75 (4.5)	53 (13.5)		4.55 (3.02–6.84)	<0.001 *
rs7534676*Alcohol			<0.001		
TT/TC*Non-Alcohol	253 (15.4)	16 (4.7)		Ref.	Ref.
CC*Non-Alcohol or TT/TC*Alcohol	1191 (72.4)	210 (61.2)		2.79 (1.65–4.72)	<0.001 *
CC*Alcohol	201 (12.2)	117 (34.1)		9.20 (5.29–16.02)	<0.001 *
rs7554974*Alcohol			<0.001		
CC/CT*Non-Alcohol	259 (15.6)	19 (5.4)		Ref.	Ref.
TT*Non-Alcohol or CC/CT*Alcohol	1195 (72.2)	212 (60.7)		2.42 (1.48–3.94)	<0.001 *
TT*Alcohol	201 (12.1)	118 (33.8)		8.00 (4.77–13.44)	<0.001 *
rs7518631*Alcohol			<0.001		
GG/GA*Non-Alcohol	260 (15.7)	19 (5.4)		Ref.	Ref.
AA*Non-Alcohol or GG/GA*Alcohol	1194 (72.1)	212 (60.7)		2.43 (1.49–3.96)	<0.001*
AA*Alcohol	201 (12.1)	118 (33.8)		8.03 (4.78–13.49)	<0.001 *
rs72936453*Alcohol			<0.001		
AA/AT*Non-Alcohol	260 (15.7)	19 (5.4)		Ref.	Ref.
TT*Non-Alcohol or AA/AT*Alcohol	1194 (72.1)	212 (60.6)		2.43 (1.49–3.96)	<0.001 *
TT*Alcohol	201 (12.1)	119 (34.0)		8.10 (4.83–13.60)	<0.001 *
rs17111068*Alcohol			<0.001		
GG/GT*Non-Alcohol	178 (10.7)	4 (1.3)		Ref.	Ref.
TT*Non-Alcohol or GG/GT*Alcohol	1265 (75.7)	196 (63.4)		6.90 (2.53–18.79)	<0.001 *
TT*Alcohol	228 (13.6)	109 (35.3)		21.27 (7.70–58.81)	<0.001 *
rs76655136*Alcohol			<0.001		
TT/TC*Non-Alcohol	167 (10.0)	6 (1.7)		Ref.	Ref.
CC*Non-Alcohol or TT/TC*Alcohol	1279 (76.5)	220 (64.0)		4.79 (2.09–10.95)	<0.001 *
CC*Alcohol	225 (13.5)	118 (34.3)		14.60 (6.28–33.96)	<0.001 *
rs10778364*Alcohol			<0.001		
CC/CT*Non-Alcohol	586 (35.5)	76 (20.6)		Ref.	Ref.
TT Non-Alcohol or CC/CT*Alcohol	915 (55.4)	203 (55.0)		1.71 (1.29–2.27)	<0.001 *
TT*Alcohol	152 (9.2)	90 (24.4)		4.57 (3.21–6.50)	<0.001 *
rs34200934*Alcohol			<0.001		
GG/GT*Non-Alcohol	365 (22.4)	42 (11.0)		Ref.	Ref.
TT*Non-Alcohol or GG/GT*Alcohol	1086 (66.7)	236 (61.9)		1.89 (1.33–2.68)	<0.001 *
TT*Alcohol	177 (10.9)	103 (27.0)		5.06 (3.39–7.55)	<0.001 *
rs11636446*Alcohol			<0.001		
AA/AC*Non-Alcohol	321 (19.3)	36 (9.5)		Ref.	Ref.
CC*Non-Alcohol or AA/AC*Alcohol	1150 (69.0)	228 (60.3)		1.77 (1.22–2.57)	0.003 *
CC*Alcohol	195 (11.7)	114 (30.2)		5.22 (3.44–7.89)	<0.001 *
rs79071218*Alcohol			<0.001		
TT/TC*Non-Alcohol	170 (10.2)	7 (1.9)		Ref.	Ref.
CC*Non-Alcohol or TT/TC*Alcohol	1264 (75.6)	224 (60.9)		4.30 (1.99–9.29)	<0.001 *
CC*Alcohol	237 (14.2)	137 (37.2)		14.04 (6.41–30.77)	<0.001 *
rs1975431*Alcohol			<0.001		
AA/AC*Non-Alcohol	169 (10.1)	7 (1.9)		Ref.	Ref.
CC*Non-Alcohol or AA/AC*Alcohol	1265 (75.7)	223 (60.8)		4.26 (1.97–9.19)	<0.001 *
CC*Alcohol	237 (14.2)	137 (37.3)		13.96 (6.37–30.59)	<0.001 *
rs28829404*Alcohol			<0.001		
CC/CT*Non-Alcohol	163 (9.8)	13 (3.6)		Ref.	Ref.
TT*Non-Alcohol or CC/CT*Alcohol	1287 (77.0)	218 (60.4)		2.12 (1.19–3.80)	0.011 *
TT*Alcohol	221 (13.2)	130 (36.0)		7.38 (4.03–13.51)	<0.001 *

Abbreviations: OR: odds ratio; CI: confidence interval. ORs were calculated by univariate analysis. ^a^ chi-square test; ^b^ logistic regression. * *p* value < 0.05.

## Data Availability

The data presented in this study are available on request from the corresponding authors. The data are not publicly available due to ethical considerations.
